# Primers and probe design and precision assessment of the real time RT-PCR assay in Coxsackievirus A10 and enterovirus detection

**DOI:** 10.1016/j.dib.2017.04.035

**Published:** 2017-04-28

**Authors:** Jingfang Chen, Rusheng Zhang, Xinhua Ou, Dong Yao, Zheng Huang, Linzhi Li, Biancheng Sun

**Affiliations:** Laboratory of Microbiology, Changsha Center for Disease Control and Prevention, Changsha, China

**Keywords:** Real time RT-PCR, Coxsackievirus A10, Enterovirus

## Abstract

This data article contains data related to the research article entitled “Rapid detection of enterovirus and Coxsackievirus A10 by a TaqMan based duplex one-step real time RT-PCR assay” (Chen at al., 2017) [1]. Primers and probe sequence design are among the most critical factors in real-time polymerase chain reaction (PCR) assay optimization. Linearity, sensitivity, specificity and precision are the crucial criteria which are used to evaluate the performance of a new method. This data article report the primers and probe design and precision assessment of the new assay. VP1 gene of Coxsackievirus A10 (CV-A10) and 5′-NCR of different enterovirus (EV) serotypes were retrieved from GenBank database and aligned. The intra- and inter-assay variation were assessed using high, medium and low concentration of control plasmid DNA and viral RNA samples.

**Specifications Table**

**Table:** 

Subject area	Biology
More specific subject area	Molecular Biology, real time RT-PCR
Type of data	Table, figure
How data was acquired	In silico analysis of gene sequences using online bioinformatics tools and MEGA 5.2 software; Precision assay acquired by analysis of the threshold cycle value of control and clinical samples.
Data format	Raw, analyzed
Experimental factors	Gene sequences were retrieved from GenBank database; Standard plasmid DNA were constructed; Plasmid DNA and viral RNA concentration were quantified and the genome copies were calculated
Experimental features	Primers and probe were designed using Primer Express software (version 3.0; Applied Biosystems) and assay precision were determined by real time RT-PCR
Data source location	Changsha, China
Data accessibility	Data with this and the main article

**Value of the data**

• Rapid detection is crucial for Coxsackievirus A10 control along with the increasing circulation worldwide in the recent years.
• Specific and conserved regions for primers and probe design are defined using multiple sequence alignment.
• The intra- and inter-assay reproducibility are assessed using different concentrations of plasmid DNA and viral RNA.

## 1 Data

Using TaqMan probes we have previously established a real time RT-PCR method for Coxsackievirus A10 and other enterovirus detection [1].

**Fig. 1:**
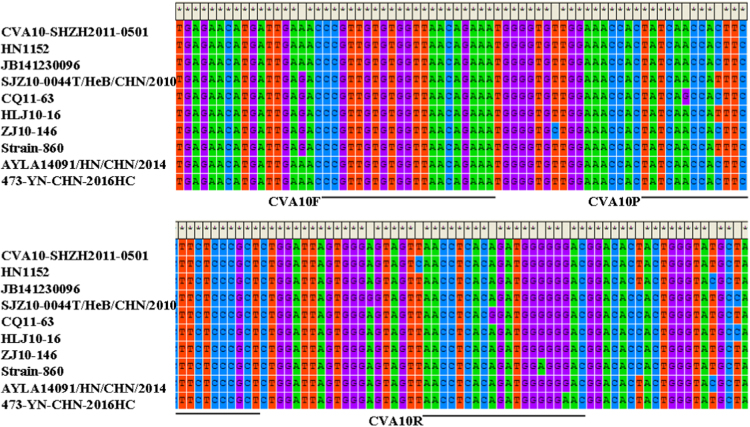
Multiple alignment of vp1 gene of various CV-A10 isolates. (CVA10-SHZH2011-0501, Acession #JX473446; HN1152, Acession #JX947811; JB141230096, Acession #KC867039; SJZ10-0044T/HeB/CHN/ 2010, Acession #KF246671; CQ11-63, Acession #KF999731; HLJ10-16, Acession #KF999744; ZJ10-146, Acession #KF999786; Strain-860, Acession #KM048110; AYLA14091/HN/CHN/2014, Acession #KU885560; 473-YN-CHN-2016HC, Acession #LC167417); Positions and sequence of the developed primers (CVA10F, CVA10R) and probe (CVA10P) are indicated.

**Fig. 2:**
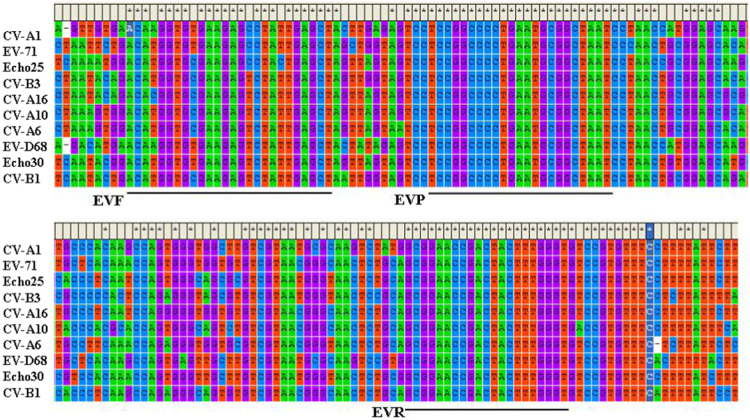
Multiple alignment of 5′NCR of various enterovirus. (CV-A1, Acession #AF499635; CV-A6, Acession #KJ541168; CV-A10, Acession #KP289402; CV-A16, Acession #JF738004; EV-71, Acession #HQ647178; CV-B1, Acession #KP260537; CV-B3, Acession #M88483; Echo25, Acession #KX139460; Echo30, Acession #KC897073; EV-D68, Acession #KT825142); Positions and sequence of the developed primers (EVF, EVR) and probe (EVP) are indicated.

The data presented in this article show the conserved regions for primers and probe design using multiple sequence alignment (Fig. 1, Fig. 2). Table 1 represents data of the average threshold cycle (Ct) value, standard deviation (SD) and the coefficient of variability (CV) of different concentrations of plasmid DNA and viral RNA.

## 2 Experimental design, materials and methods

### 2.1 Primers and probes design

Sequences of CV-A10 VP1 gene and 5′-NCR of different enterovirus serotypes were downloaded from NCBI (http://www.ncbi.nlm.nih.gov/) database. Multiple sequences alignment was performed using the Clustal W algorithm in MEGA 5.2 software. Primer Express software (version 3.0; Applied Biosystems) was used to design the primers and probe for CV-A10 assay based on their highly conserved and specific regions (Fig. 1). For EVs detection, the primers and probe were chosen according to previous studies [2], [3]; A small amplicon of 142 nucleotides in size were produced (Fig. 2). All oligonucleotides were synthesized by TAKARA (Dalian, China).

### 2.2 Standard plasmid DNA construction

To facilitate viral quantification, plasmids containing the target genes were constructed. The targeted gene segments were amplified and purified, and then cloned into the pMD-18 Vector by using the T-A clone kit (TaKaRa, Dalian, China). Constructed plasmid containing amplified segments were purified and sequenced in both directions. The standard plasmid concentration were quantified and the genome copies were calculated by as follows: copy number=[(plasmid concentration)/(molarmass)]×(6.02×10^23^).

### 2.3 Repeatability and reproducibility of the assay

To assess the intra- and inter-assay reproducibility, 2.0×10^6^, 2.0×10^3^ and 2.0×10^1^ of plasmid DNA were diluted; Viral RNA from a CV-A10 clinical sample was extracted using the QIAamp viral RNA mini kit (Qiagen, Hilden, Germany) according to the manufacturer׳s instructions and the equivalent RNA copies were calculated. The intra-assay variation was assessed with the samples in triplicate and the inter-assay variation was determined by three independent runs. The reproducibility was then analyzed based on the standard deviation (SD) and the coefficient of variability (CV) of the Ct average (Table 1).

## Figures and Tables

**Table 1:** Intra- and inter-assay variations in different concentrations of plasmid DNA and viral RNA.

Copy number	Intra-assay variation			Inter-assay variation		
	Mean Ct	SD	%CV	Mean Ct	SD	%CV
duplex rRT-PCR of EV assay						
Plasmid DNA						
2.0×10^6^	19.31	0.14	0.74	19.28	0.42	2.19
2.0×10^3^	29.18	0.09	0.33	29.36	0.46	1.57
2.0×10^1^	36.16	0.37	1.02	36.26	0.95	2.62
Viral RNA						
2.5×10^7^	20.59	0.02	0.11	20.22	0.31	1.26
1.0×10^6^	23.02	0.09	0.39	22.78	0.39	1.72
4.0×10^4^	25.65	0.06	0.24	25.19	0.41	1.64
				
duplex rRT-PCR of CV-A10 assay						
Plasmid DNA						
2.0×10^6^	20.18	0.21	1.04	19.63	0.37	1.88
2.0×10^3^	29.77	0.19	0.63	29.19	0.61	2.09
2.0×10^1^	37.09	0.59	1.58	36.19	0.49	1.34
Viral RNA						
2.5×10^7^	19.74	0.03	0.13	19.89	0.28	1.39
1.0×10^6^	22.14	0.06	0.28	22.45	0.35	1.57
4.0×10^4^	24.54	0.08	0.31	24.82	0.34	1.37

## References

[1] Chen J.F., Zhang R.S., Ou X.H., Yao D., Huang Z., Li L.Z., Sun B.C. (2017). Rapid detection of enterovirus and Coxsackievirus A10 by a TaqMan based duplex one-step real time RT-PCR assay. Mol. Cell. Probes.

[2] Dierssen U., Rehren F., Henke-Gendo C., Harste G., Heim A. (2008). Rapid routine detection of enterovirus RNA in cerebrospinal fluid by a one-step real-time RT-PCR assay. J. Clin. Virol..

[3] Pabbaraju K., Wong S., Wong A.A., Tellier R. (2015). Detection of enteroviruses and parechoviruses by a multiplex real-time RT-PCR assay. Mol. Cell. Probes.

