# Supplementary material for: Primers and probe design and precision assessment of the real time RT-PCR assay in Coxsackievirus A10 and enterovirus detection
Source: Data Brief. 2017 Apr 28;12:418–22. doi: 10.1016/j.dib.2017.04.035 (PMC5426041; doi:10.1016/j.dib.2017.04.035)
Supplement: Supplementary file 1 — Supplementary material [file mmc1.doc]

Dear editor:

We assure the content of this paper has been neither published elsewhere and all authors have contributed significantly to this study and all of them are in agreement with the submission. We declare there is no conflict of interest.

Biancheng Sun
